# Acute Schmallenberg Virus Infections, France, 2012

**DOI:** 10.3201/eid1902.121281

**Published:** 2013-02

**Authors:** Corinne Sailleau, Emmanuel Bréard, Cyril Viarouge, Alexandra Desprat, Virginie Doceul, Estelle Lara, Jérôme Languille, Damien Vitour, Houssam Attoui, Stéphan Zientara

**Affiliations:** Author affiliations: Agence Nationale de Sécurité Sanitaire, Maisons-Alfort, France (C. Sailleau, E. Bréard, C. Viarouge, A. Desprat, V. Doceul, E. Lara, D. Vitour, S, Zientara);; Direction Générale de l’Alimentation, Paris, France (J. Languille);; Institute for Animal Health, Pirbright, UK (H. Attoui)

**Keywords:** Schmallenberg virus, viruses, epidemiology, overwintering, acute infections, Orthobunyavirus, cattle, sheep, goats, expedited, France

**To the Editor:** After unexpected emergence of bluetongue virus serotype 8 in northern Europe in 2006 (*1*), another arbovirus, Schmallenberg virus (SBV), which is transmitted by *Culicoides* spp. biting midges, emerged in Europe in 2011 and caused disease outbreaks among ruminants (*2*). Nonspecific clinical signs such as fever, decreased milk production, and diarrhea were associated with acute infection in cattle, and late abortions and birth defects in newborns were associated with infection of pregnant cows, ewes, and goats (*2**,**3*).

SBV, which belongs to the family *Bunyaviridae* and genus O*rthobunyavirus*, was detected in Germany, the Netherlands, and Belgium in 2011. This virus was later detected in the United Kingdom, France, Italy, Luxembourg, Spain, Denmark, and Switzerland (*4*). As of August 1, 2012, a total of 5,701 infected farms were reported in Europe (2,498 sheep farms, 3,124 cattle farms, and 79 goats farms) (www.survepi.org/cerepi/). France has been the country most affected: it had 2,650 SBV-infected farms (*5*), (i.e., in which >1 malformed offspring was positive for SBV by real-time reverse transcription PCR [RT-PCR] on 1,128 sheep farms, 1,505 cattle farms, and 17 goat farms).

Abnormalities detected in offspring in 2011 and in early 2012 were caused by infections acquired in 2011 (*4*). At that time, it was unclear whether SBV could survive the 2011–2012 winter and remain a threat to Europe. We report data suggesting that SBV overwintered or was reintroduced in France.

On May 16, 2012, a herd of 75 dairy cows in southwestern France (Pyrénées-Atlantiques) had hyperthermia and decreased milk production. Of 18 cows tested by the Agence Nationale de Sécurité Sanitaire (Maisons-Alfort, France), 9 were positive for SBV by PCR (*6*) (cycle threshold [C_t_] range 17–36.5) and negative for SBV by ELISA (IDVet, Montpellier, France), 1 was positive by PCR and ELISA, and 8 were negative by PCR and ELISA (Figure). 

**Figure:**
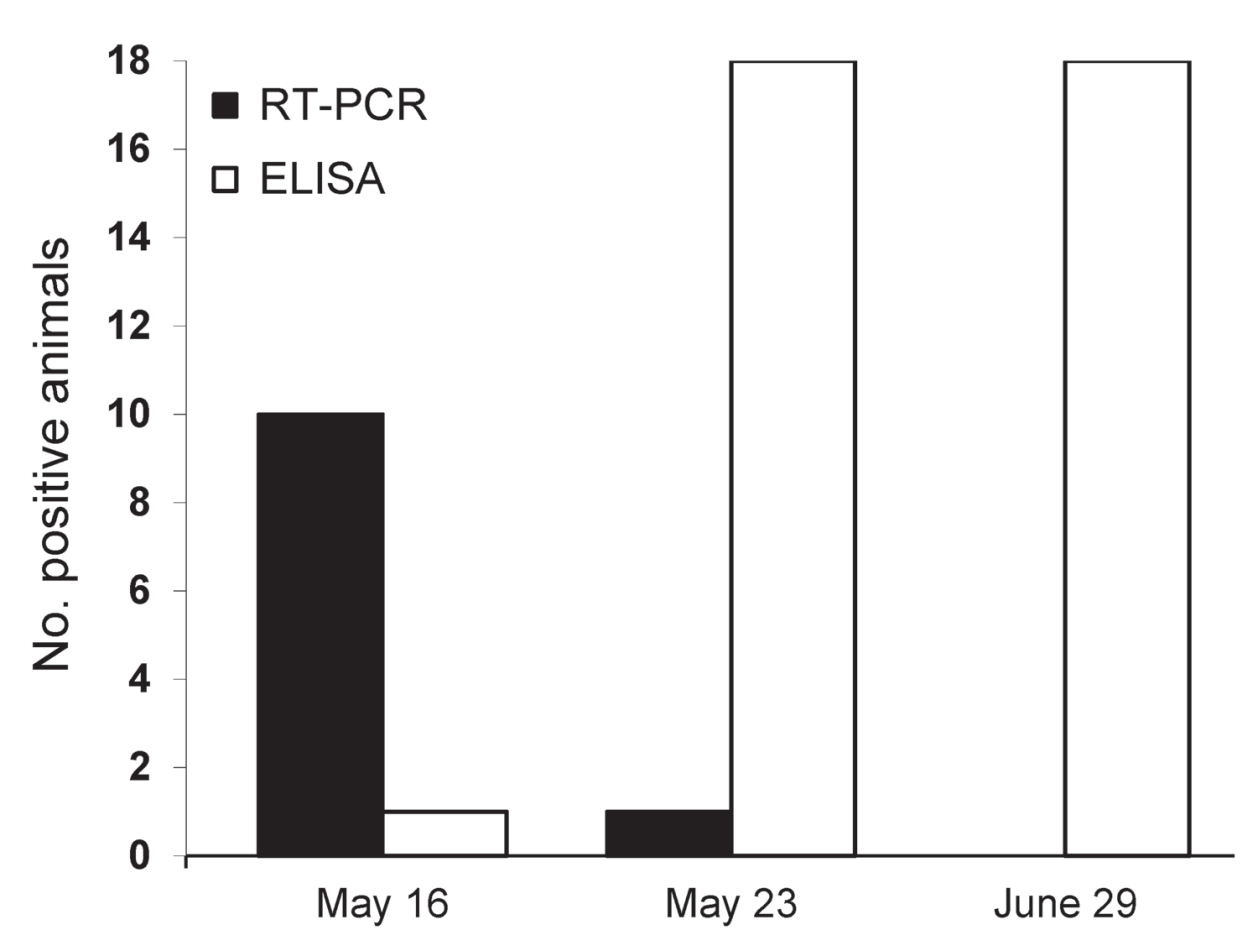
Number of cows positive for Schmallenberg virus according to reverse transcription PCR (RT-PCR) and ELISA, France, 2012.

On May 23, a week after the first samples were collected, all cows tested were positive by ELISA and only 1/18 cows were positive by RT-PCR. On June 29, all 18 cows tested were negative by RT-PCR. Detection of SBV-neutralizing antibodies by virus neutralization assay in serum samples obtained on May 23 confirmed SBV ELISA results and showed that a commercial IgG ELISA is suitable for detection of acute cases of SBV.

Viremia, as measured by RT-PCR, occurs during the first 5 days after acute infection (*2*). Antibody response against SBV, as measured by ELISA, is detected during or after the first 10 days after experimental infection (C. Sailleau et al., unpub. data). Accordingly, serologic and molecular data showed that acute SBV infection occurred in cattle in southwestern France in May 2012, suggesting that SBV overwintered or was reintroduced. Moreover, in July 2012, another case of acute SBV infection was identified in Finistère (Brittany). A cow with hyperthermia and diarrhea was SBV positive by RT-PCR (C_t_ 31) and negative by ELISA, which indicated a recent SBV infection.

Three blood samples (each 170 µL) from RT-PCR–positive cows (C_t_ range 15–21) (1 from the Finistère and 2 from Pyrénées-Atlantiques) were injected into 3 adult IFNAR^−/−^ mice. Seventy microliters of each sample was injected intraperitoneally and 100 μL was injected into the neck scruff of the same mice. After 4 days, the mice showed no clinical signs and their weights were unchanged. Blood samples were collected and tested for SBV by RT-PCR. All mice were positive for SBV RNA (C_t_ range 18–31). These data showed that blood samples from cows contained virus RNA and confirmed that SBV reemerged in 2012.

On July 25, 2012, SBV infection was identified in a cow in Jura Canton in the northwestern, French-speaking region of Switzerland (Romandie) (*7*). A serologic study conducted in the United Kingdom showed that several cattle and sheep seroconverted for SBV in 2012 (*8*). However, our data show that SBV survived the winter, when midge numbers decreased. The precise mechanisms of SBV overwintering are not known and need to be explored.

The consequences of SBV recirculation should be investigated, particularly in pregnant cows, ewes, and goats. The 2 SBV-positive farms described in this report are located in a previously SBV-free area (Finistère-Brittany) or an area in which the infection rate was low (Pyrénées-Atlantiques) in the winter of 2011–2012, during which seroprevalence for most herds was probably weak (C. Sailleau et al., unpub. data). Therefore, reemergence of cases of congenital forms of SBV infection in France and others areas of Europe can be expected.
